# The Harrow Cottage Hospital: Its Architecture and Its Critics

**Published:** 1914-05-02

**Authors:** Paul Waterhouse


### THE HARROW COTTAGE HOSPITAL.

### Its Architecture and its Critics.

REPORTED UPON BY MR. PAUL WATERHOUSE, M.A., F.R.I.B.A.

We have received from Mr. Paul Waterhouse, F.R.I.B.A., a communication on the subject of this building. Mr. Waterhouse, being from time to time engaged in hospital construction, visited the hospital recently as a matter of personal interest, and, being acquainted with the report published in our issue of February 21, sends us his notes as a comment upon our criticisms. Incidentally, they are also a comment upon the speches made by Mr. Stuart and others at the annual meeting, a report of which appeared in the *Harrow Observer* of March 7. Generally, it may be pointed out that Mr. Waterhouse's report confirms the accuracy of the criticisms that Sir Henry Burdett made, and enforces the point that Mr. Stuart was wrong in reference to the staircases, seeing that steps have to be ascended when carrying a patient from the main entrance to the ward.

Mr. Waterhouse, as an architect, is perhaps naturally less severe on certain architectural aspects of the case than we had thought it right to be. He says: "The building, a typical specimen of the work of Mr. Arnold B. Mitchell, is undeniably attractive. It exhibits on the north or road frontage strong characteristics of that type of English Renaissance work which is associated with Mr. Mitchell's authorship, and the only legitimate criticisms against its good looks are (1) that, unless money was unusually plentiful, too much would seem to have been spent on such a feature as the masonry of the front doorway; and (2) that the treatment of the roofs, though unquestionably picturesque, has produced on the upper floor a disposition of space which is at once wasteful and cramping. In fact the lumber-rooms are exceptionally spacious, and some of the apartments are unduly compressed."

The above remarks, in spite of this measure of praise, constitute a confirmation of our attack on certain external and internal shortcomings. We were, it seems, technically in error in our use of the term "Gothic," but we feel justified in repeating the substance of our objection to the exterior—viz. its subordination of hygienic needs to display—though we realise that Mr. Waterhouse does not and would not put it as strongly as this. Moreover, the remarks made by him on the upper floor are confirmatory in a large degree of our own observations on this very point.

Mr. Waterhouse, describing the interior (of which he commends, as we did, the wards themselves and some parts of their immediate accessories), says: "With great architectural ingenuity and some measure of success the designer has obtained symmetry on the main front by making the window of the operating-room a facsimile of that which lights the sisters' sitting-room; obviously this is a matter of compromise. It means that the window of the latter is somewhat too large, and that of the former something too small, but the result, in spite of the twofold sacrifice, is far better than one would expect." "Personally," Mr. Waterhouse adds—and we agree with him—"I do not think that in a matter like the lighting of an operating-room any consideration should interfere with the strict scientific necessities of the case."

Continuing, Mr. Waterhouse says: "There are one or two matters which Mr. Mitchell would probably do otherwise if he were doing them again. The service lift is obviously not what it ought to be, and the 'cut-offs' of the sanitary blocks are not true 'cut-offs,' for the bed-pan washing sink is, in each case, placed in the 'cut-off' instead of being as much isolated as the w.c." He adds: "You were quite correct in implying that there are steps up to the wards. It is true that at the east end, owing to the rise in the ground, a patient can be brought into the building and placed in a ward without ascending any steps, except the one (or two) at this entrance. But at the main entrance there are two steps by the door and five inside (seven in all). I understand that for this reason the east entrance is the one used for admitting casualty cases."

"The lack of a ground-floor sitting-room or office for the sister-in-charge is also a fair criticism. The ground-floor sitting-room is used by all the nursing staff, and is intended to be combined with the adjoining waiting-room (by removal of a partition) to form a committee room when required."

"The private room of the sister-in-charge is one of the upstairs rooms, which, it is true, has a beautiful view, but is rather inaccessible."

"In conclusion," says our correspondent, "I hope that these observations may be of some use in clearing up what appears to have become a kind of controversy between the hospital and its critics, and I should like to take the opportunity in your columns of acknowledging the courtesy with which I was shown round the building, which, however much it may deserve some of your criticisms, is, without doubt, one of considerable charm and amenity."

